# Gastric canthariasis caused by invasion of mealworm beetle larvae in weaned pigs in large-scale farming

**DOI:** 10.1186/s12917-020-02657-0

**Published:** 2020-11-11

**Authors:** Remigiusz Gałęcki, Mirosław Mariusz Michalski, Karol Wierzchosławski, Tadeusz Bakuła

**Affiliations:** 1 Department of Veterinary Prevention and Feed Hygiene, Faculty of Veterinary Medicine, University of Warmia and Mazury in Olsztyn, Oczapowskiego 13st, 10-718 Olsztyn, Poland; 2 Department of Parasitology and Invasive Diseases, Faculty of Veterinary Medicine, University of Warmia and Mazury in Olsztyn, Oczapowskiego 13st, 10-718 Olsztyn, Poland; 3 Private Veterinary Practice Agrobiovet, Orcholska 61st, 62-200 Gniezno, Poland

**Keywords:** Coleoptera, Tenebrionidae, *Tenebrio molitor*, Insecta, Weaning pigs, Storage pest

## Abstract

**Background:**

Mealworm beetle *T. molitor* (Coleoptera: Tenebrionidae) (Linnaeus, 1758) is one of the most important cosmopolitan primary storage pests, scavenging on a variety of post-harvest grains and affecting the quality and safety of food and feed. In addition to being an important factor in feed hygiene, the insect can also be an epidemiological factor of canthariasis. Livestock infestations with *T. molitor* are rarely reported. This article describes *T. molitor*-caused canthariasis in pigs in large scale closed-cycle farming.

**Results:**

In the spring, we registered a significantly increased mortality among weaned pigs. In autopsy, live 3–6 mm long *T. molitor* larvae were found in their stomachs, especially in the non-glandular oesophageal region, on average 2–3 larvae per 10 cm^2^ of gastric mucosa. Corrective actions reduced the number of deaths back to basal levels.

**Conclusions:**

This is the first documented case of potentially lethal gastric canthariasis in weaned pigs, caused by invasion of *T. molitor* larvae. Although canthariasis caused by *T. molitor* has not been a significant problem in farm animals so far, our case indicates that the presence of mealworm beetles is a potential threat to animal welfare and health.

## Background

Worldwide increase of meat consumption and animal production causes growing demand for plant-derived feed. In Poland, the production of industrial feed has been initially expected to amount to almost 12 million tons in 2019 [1]. Obviously, the nutritional value of feed is the paramount factor in proper development and fast growth of farm animals [2], but feed hygiene and biological safety are not less important. Storage pests are among the most significant biotic threats to agricultural yield and safety. Approximately 5–10% of grain are lost during storage due to toxins from microflora and direct damage by pest insects. Grain insects are generally divided into primary (attacking whole, unbroken grains) and secondary (infesting damaged grain, milled products and dust). Adult insects can survive for up to 3 years, and 4 or even 6 generations can develop under favorable conditions on heated premises [3]. One of the secondary pests, the mealworm beetle (*Tenebrio molitor*) is a common cosmopolitan pest in European grain warehouses, feed factories, mills, etc., scavenging on a variety of post-harvest grains. It is one of the largest beetles (up to 15.5 mm) with the complete metamorphosis (egg, larva, pupa, imago) cycle lasting from 19 to 31 weeks, depending on temperature and humidity. Mealworm beetle females lay 300–400 eggs, larvae (approx. 2 mm) hatch within 5–12 days and reach approx. 1 cm in 4–6 weeks, the larval period varies from 22 to 100 days, and the pupae period takes about 8 days [4].

Insects are now a legitimate feed ingredient, and insect market for animal feed is increasing around the world. In this context, *T. molitor* deserves special interest since it has been reported to possess a high nutritional value and to be an acceptable protein source for poultry [5] and other farm animals. However, due to its release of mutagenic carcinogens (benzoquinones) this beetle is particularly dangerous to human and animal health [6]. Respiratory allergies may develop upon prolonged contact with products infested by *T. molitor* [7] and the beetle can be an epidemiological factor of canthariasis [8].

Currently, in human and veterinary medicine, canthariasis is defined as the invasion of beetle larvae on a living organism, during which, at least for some time, the larvae feed on dead or living host tissues, on body fluids or the food consumed by the host [9]. Insects live in the host from the first to the third stage of their development, and then imagoes leave the organism to complete the life cycle. Developmental forms often enter the animal’s body together with feed, so zootechnicians and veterinarians must be vigilant and take necessary precautions if adult beetles or any traces of the insects are present on farm premises. Larvae adapt to a particular environment, they enter the hypobiosis state both inside and outside the host [10]. The diversity of pathological changes and clinical signs can be explained by natural behavioral differences between insect species [10]. Therefore, clinical manifestations of canthariasis vary greatly depending on the entry site of the invasion, the insect species, the number of larvae and their target tissue(s). It should be remembered that canthariasis may, at least initially, not give clear clinical symptoms [11, 12]. The main categorization of the disease relies on anatomical reference to the invasion location in the host [9, 13]. The larvae can locate in eye orbits, that causes foreign body sensations in the host’s eye or nasopharynx. Accidentally ingested larvae or eggs may remain in the mouth, thereby causing damage to the gums and dental spaces. Skin canthariasis can occur in various forms, such as furunculi, creeping canthariasis (larvae nesting in the fur or under the feathers) or subcutaneous canthariasis, caused, for example, by a lesser mealworm [14]. Dermatologic symptoms include boils, pruritus, erythema, and severe pain caused by the movement of larvae in the skin and in subcutaneous tissues [9, 15]. During the invasion, a polymorphic inflammatory infiltrate may be observed in neighboring tissues, which consists mainly of lymphocytes and neutrophils, with addition of eosinophils, fibroblasts, histiocytes, basophils, mast cells, plasma cells and Langerhans cells [16]. The inflammatory reaction leads to the activation of mast cells and the production of IgE, which can limit the development of larvae [16]. The most frequently reported complication in this disease is a secondary bacterial infection. Gastric canthariasis, caused by swallowed eggs or larvae, manifests as nausea and vomiting, stomach ache and abdominal bloating, loss of appetite and weight loss, or diarrhea resembling intestinal parasite infection [12]. In extreme cases, the larvae penetrate through the wall of the digestive tract and invade other organs; this, however, is rare, because most die before reaching the small intestines. Untreated canthariasis may lead to death of the animal as a result of anaphylactic shock, intoxication or secondary bacterial infection of damaged host tissues. Canthariasis caused by *T. molitor* is very rarely reported.

This article describes the first case of potentially lethal gastric canthariasis caused by *T. molitor* in young pigs (*Sus scrofa domesticus*) in large-scale farming.

## Results

On the farm, the average long-term year-to-year mortality rates by production group was: piglets 13.5%, weaned pigs, including those aged 4–10 weeks, 2.5%, porkers 3.1% and sows 3.2%. Then, in the spring, about 2 weeks after weaning and switching to the Starter feed, the number of deaths exceeded 3.5%. Only weaned pigs experienced an increase of mortality. Necropsies (n-15) revealed not only colibacillosis typical for weaned pigs, but also pronounced hemorrhagic gastritis in all individuals. The contents of the digestive tract were blood-colored. The intestinal mucosa and mesentery vessels were severely hyperemic. No other gross lesions were observed. In the gastric contents, especially in the oesophageal region (non-glandular part), numerous live 3–6 mm beetle larvae were found, average 2–3 specimens per 10 cm^2^ of gastric mucosa. Sudden deaths of weaned pigs was observed as major clinical sign. Individuals have had characteristic signs of edema disease (colibacillosis) such as neurological symptoms (ataxia and convulsions) and subcutaneous edema (i.e. swelling of the eyelids). Unfortunately, these findings were not documented photographically. No other clinical diseases were found during clinical examination of live animals and in post-mortem studies. An average daily weight gain was 0.42 kg/day for weaned pigs and 0.89 kg/day for porkers.

**Fig. 1:**
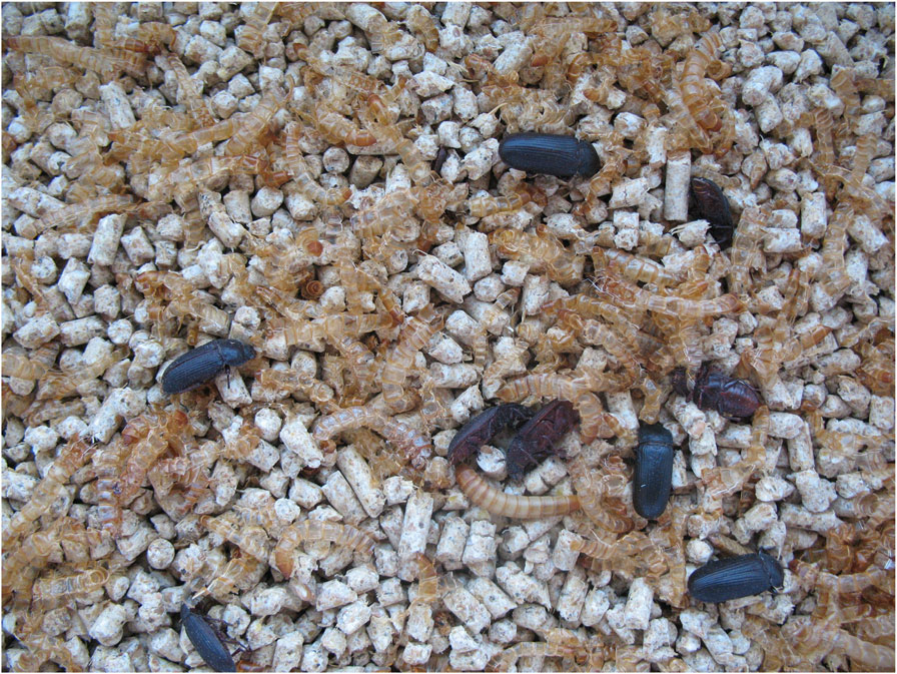
Larva and adults of mealworm beetle (*Tenebrio molitor*) obtained from feed, after 3 months incubation period

**Fig. 2:**
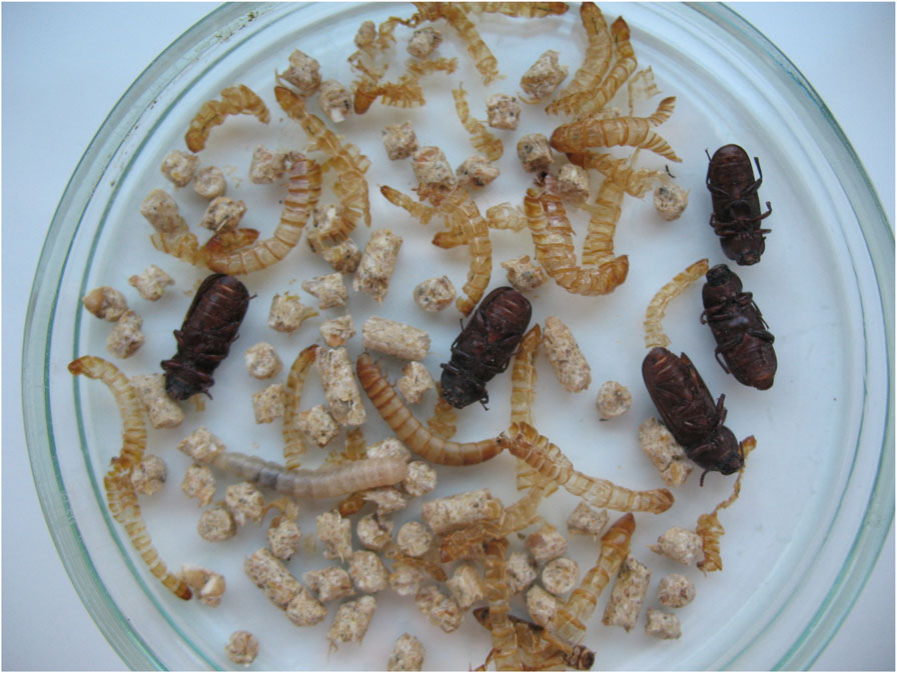
Larvae and adults of mealworm beetle (*Tenebrio molitor*) obtained from stomach, after 2.5 months incubation period

Upon inspection of the feed, eggs of average size 1.7 × 0.7 mm were observed. Corrective actions were taken immediately: thorough cleaning and washing of troughs and changing the young animals’ feed. Also, emptying and mechanical cleaning of feed residue from automatic feeding system was carried out. Feed components from a different source were used and the formulation remained unchanged. Virkon S (Bayer AG, Leverkusen, Germany) was used for disinfection according to the manufacturer’s instructions. These measures quickly reduced the number of deaths back to basal levels. Also, post-mortem examination of the animals which died after the corrective actions did not reveal the presence of beetle larvae. No colibacillosis symptoms were observed after corrective actions. Feed contaminated with development forms of *T. molitor* was disposed of in accordance with the recommendations and time requirements of the General Veterinary Inspectorate. Further laboratory observation of the contaminated feed revealed the first larvae hatching within 2 weeks, and adult *T. molitor* beetles within 3 months. Larvae obtained from the stomach developed into adult stage after 2.5 months of incubation Figs. 1 and 2.

## Discussion

This case indicates that *T. molitor*, whether as a storage pest or as a feed additive in the form of unprocessed mealworm larvae poses direct, potentially lethal, threat to farm animals. However, *T. molitor* larvae are often added to feed as an alternative protein source. Dried and grounded larvae are usually used in the studies on the pig’s nutrition [17, 18]. Mealworm larvae closely matched with fish meal, making it a potentially attractive alternative protein-rich feed ingredient for livestock feed industry [17]. Supplementation of dried mealworm larvae up to 6% in weaning pigs’ diet may improve growth performance and nutrient digestibility without any detrimental effect on immune responses [5]. Ao and Kim demonstrated, that dried mealworm powder can replace fish meal in weaning pigs’ diet without any negative effect on growth performance or nutrient digestibility [18]. Mealworm larvae hydrolysate had higher digestibility compared fermented poultry by-product or hydrolyzed fish soluble meal [19]. Our results warn against using live larvae, in particular for weaned pigs, since weaning immunosuppression could facilitate the infestation. Only processed, dried *T. molitor* larvae meal will not pose a direct threat to animal health.

We are not able to fully determine how the larvae get into the stomach. Suggesting the size of insects in the stomach, we think that pigs could swallow already hatched larvae. Probably a feed contaminated with eggs had been put into the silo. There, due to unfavorable conditions (low temperature and humidity), larvae did not hatch from eggs. It is possible that the feed was some time outside the silo before animals feeding (e.g. after mixing with water or it was left in the troughs) and during this time the larvae were probably released from eggs. Then mealworm larvae (approx. 2 mm) could be swallowed by pigs. This explains the results in which we found the eggs in the feed obtained from the silo and the live larvae in the stomach. The main problem is the lack of mechanical disruption of the larvae, due to a feeding style (liquid feeding regime) that prevents chewing on them, and the small size of the first larvae. In the stomach we found larvae of different sizes, which indicates that they were of different ages. Assuming that the animals were fed for 2 weeks with contaminated feed, some of the larvae could be digested and some could be located in a non-glandular region by active movement.

According to literature reports, *T. molitor* may pose a health risk. In many cases, eggs or larvae are eaten by humans and animals along with grain-based food. Usually they are digested or they passed along with the digestive tract and excreted with feces. In some cases, however, they are able to survive and live in alimentary tract. The first reports about the presence of insect larvae in human organs date back to the 19th century, when they were observed in the tonsils, nose and bladder, or in the umbilical cord [20, 21]. Insects, including *T. molitor* larvae were diagnosed in the gastrointestinal tract of humans, including the stomach and the intestines [22–24]. Hinman and Faust [25] described the occurrence of *T. molitor* larvae in human organs. They found that the gastrointestinal tract is the most common habitat for parasitizing larvae of this beetle [25], which was also confirmed by Palmer’s research [8]. Rodriguez-Morales et al. [26] reported ulcer infestation of *T. molitor* in an AIDS patient. The last human case of canthariasis caused by *T. molitor* was described in 2019 by Aelami et al. [27] and concerned urinary canthariasis due to mealworm larva in a 10 years old boy in Iran. There are few reported cases of live mealworm beetle larvae in the body of animals. In 2013, a case of *T. molitor* larvae development in a black-legged heron under the skin in the area between the esophagus and the trachea was described [28]. Gastrointestinal canthariasis caused by mealworm beetle larvae is much rarer. In current literature there are no such reports in the case of farm animals. It should also be noted that beetles of the Tenebrionidae family are potentially significant allergens for workers exposed to grains or grain products [29]. Therefore, the presence of these grain pests not only directly threatens animals, but also indirectly threatens the workers on farms and feed factories.

It should be remembered that there is a negligible chance of invasion of beetle larvae in animal breeding using Good Animal Husbandry Practices principles. The disease, although it may not cause clinical symptoms, can be detected by the presence of traces of pests on feed that can be perceived by the owner with the unaided eye. Control of *T. molitor* at farms is also important because of its role as a potential mechanical vector for pathogens [25, 30]. Mainly, the development of *T. molitor* results from the negligence of the animals’ owner or feed mill, therefore, in each case, comprehensive disinfestation should be carried out. The occurrence of canthariasis may indicate a lack of welfare, hygiene of animal feed or a lack of proper disinfection of feed silo. The mealworm beetles infestation on the area of the described farm in the spring period is probably correlated with the phenology of the occurrence / migration of these insects in the environment. Other predisposing factors may be bacterial infections or immunosuppression. In addition, the weaning immunosuppression could play a key role in our case. The occurrence of beetle larvae in the gastrointestinal tract could lead to irritation and mentioned gross lesions in stomach. Presence of *T. molitor* could also affect mucosal-associated lymphoid tissue (MALT) by mechanical stimulation. However, due to the post-weaning immunosuppression, tolerance of larvae, or provoked damage to the gastric mucosa may have occurred. Based on the obtained data, we are not able to determine the relationship between gastric canthariasis and cases of colibacillosis. It cannot be excluded that other pathological factors prevented digestion of the larvae. The actual problem caused by mealworms is still unclear - some gastritis appears not necessarily a reason for the problem. But the fact that correction reduced mortality is strong indication that the larvae were the problem.

The first and most important aspect of prevention of insect larvae infestation in farm animals is to ensure basic animal and feed hygiene and to maintain welfare, because poor sanitary conditions in breeding and feeding rooms are probably the most important factor determining this disease. The feed should be regularly monitored for the presence of adults whose pheromones can attract new individuals. Small / backyard farms can play an important role, where due to cost savings the correct preventive and the hygienic procedures are not applied. Such farms can be an important factor in the migration of storage pests. Forage left outside for a long time or feed residue in warehouses and silos at farms can also attract these pests.

For large farms, individual action plans should also be implemented, which include regular emptying and cleaning of the grain silos, storing of feed in appropriate containers and conditions, and control whether these operations are carried out. The use of Good Hygiene Practice, including pest control, in both livestock farming and the feed industry, can prevent many cases of the canthariasis. For a large population of *T. molitor* in farm buildings, disinfestation should be considered. These practices should be particularly used in places where infestation with these pests has previously occurred. No treatment for gastric canthariasis caused by *T. molitor* has been developed in livestock at present. Appropriate antiparasitic agents may help in the treatment of invasion. We believe that in the case of gastrointestinal canthariasis, similar therapeutic procedures can be used as in the treatment of myiasis. In countries where myiasis is endemic, systemic injection or the use of pour-on preparations to prevent flies are allowed. Numerous insecticides are available for treatment, including macrocyclic lactones (doramectin, eprinomectin, ivermectin or moxidectin) in various formulations. Doramectin and ivermectin after systemic administration have a systemic effect which may lead to the elimination of live larvae within the body. Eprinomectin is also effective in controlling the insect larvae invasion, however, in many countries it is only registered for cattle. Oral administration of avermectin may also lead to spontaneous death of larvae in the digestive tract.

## Conclusions

Although canthariasis caused by *T. molitor* has never been a serious problem in farm animals, veterinarians in their practice note the presence of this storage pest in the production and control of feed. The described case provides reason to draw attention to the fact that in pig breeding facilities, in cases of increase pigs mortality in the weaning period, steps should be taken to control the feed for contamination with development forms of grain pests. In our case, mealworm beetles invasion occurred, and its larvae got into the stomach of pigs along with the feed, and in this unusual environment they survived. Despite the presence of this insect in the environment, cases of canthariasis on farms are detected extremely rarely, which may be due to underestimation of the pathogenic potential of this grain pest. Such cases of the *T. molitor* larvae invasion may affect the physiological status of the gastrointestinal tract and may presumably increase other pathological processes. The problem might be overlooked if mortality is not exceptional. It seems appropriate to implement periodic monitoring of feed in this type of breeding facilities for possible contamination with pests, including *T. molitor* larvae. Special procedures should also be introduced in warehouses and feed silos and plants producing animal feed to minimize the chance of the *T. molitor* developmental forms occurrence in the final product. Our research suggests that further research into mealworm-induced canthariasis should focus on the impact of this insect on the host’s immune system and histopathological changes caused by its presence.

## Methods

The case described concerns a closed-cycle pig farm (breed: Polish Landrace x Polish Large White) in the vicinity of Gniezno, Greater Poland, Poland. The approximate average annual population on the farm was 700 sows, 1,450 piglets before weaning (less than 28 days old), 2,500 weaned pigs (body weight 8–30 kg) and 4,500 porkers. The farm applies the “all-in all-out” principle. No ethical/welfare authority approval was required as samples were collected post-mortem or obtained at the request of the owner for diagnostic purposes from live animals, by certified veterinarians. Study was handled according to Good Clinical Veterinary Practice and Polish veterinary regulations [31–33]. The farm met all biosecurity requirements contained in the coordinated national program of official inspection reports of the General Veterinary Inspectorate, compatible with European Union regulations. The health status of the herd was determined: negative for porcine reproductive and respiratory syndrome virus PRRS, negative for pleuropneumonia caused by *Actinobacillus pleuropneumoniae*, positive for porcine circovirus (associated) disease caused by PCV2 virus and positive for pneumonia caused by *Mycoplasma hyopneumoniae*. Immunoprophylaxis for PCV2 has been carried out for many years using the Ingelvac Circoflex vaccine (1 ml/animal, 24–28 days old, Boehringer Ingelheim, Ingelheim am Rhein, Germany). Cases of the colibacillosis occur incidentally in individual cases. To prevent *E. coli* infections, metaphylaxis using zinc oxide at a dose of 2500 ppm in Prestarter and Starter feed was performed. The sows were fed in a dry system with rationed feed depending on the physiological condition of the animals. During the 2 weeks before weaning, piglets were fed with Prestarter granulated fodder (Porcus Silver, Lira Mixing Plant, Krzywiń, Poland). The composition of this feed consists of: crude protein- 19%, crude fats- 6.5%, lactose- 6%, lysine- 1.5%, Vitamin A- 16 000 IU, Vitamin D- 2000 IU, Vitamin E- 190 mg/kg. Weaned pigs and porkers were fed with Starter type mash feed prepared and mixed on the farm containing wheat, barley, soybean meal, vitamin and mineral mix and soybean oil. Approximate nutritional value was: crude protein 17.6%, oils and crude fats 4%, Crude fiber 4.2%, lysine 1.17%, methionine 0.38%, threonine 0.75%, tryptophan 0.2%, calcium 6 g/kg, phosphorus 4.5 g/kg, sodium 2 g/kg, Vitamin A- 6500 IU, Vitamin D- 2000 IU, Vitamin E- 85 mg/kg. Acidifying preparation (Lonacid, LNB, Kiszkowo, Poland) was added during feed production according to the manufacturer’s instructions. Piglets, weaned pigs and porkers were fed *ad libitum* from the automatic feeding machines. During the first 2 weeks after weaning, the piglets received Starter feed *ad libitum*, and in addition, for solid feed intake training and intake increase, they were given separate troughs with wet feed (Starter feed mixed with water at a 1:1 ratio) 3–5 times a day. The following deworming system was implemented in the facility: sows 2–3 weeks before parturition with ivermectin, subcutaneously at a dose of 1 ml/33 kg b.w. (Biomectin 1%, Vetoquinol, Magny-Vernois, France), weaned pigs at the age of 10 weeks, once with levamisole at a dose of 25 mg/kg b.w. (Levamol 10%, Vetoquinol, Magny-Vernois, France) or fenbendazole orally at a dose of 5 mg/kg b.w. (Fenbenat 40 mg/g, PFO Vetos-Farma, Bielawa, Poland).

Due to the increase in mortality, post-mortem examinations were carried out on 15 pigs (age 6–7 weeks) at the farm. Pigs died naturally due to gastrointestinal symptoms without any human procedures prior to veterinary intervention. After the post-mortem examination, corpses were disposed by a specialized company in accordance with applicable regulations. Four samples of feed were taken randomly from the silos weighing 1 kg each. Samples were taken from the feed silo hopper manually without using a probe. The feed was stored in silos max. 7–10 days. Also discovered larvae in the stomach content were collected for further research. Samples of the Starter feed were incubated at 29.5 °C, humidity 70% in the Biological Hazard Laboratory to maintain stable conditions for possible storage pest growth. The hatched larvae (3–4 mm) were then transferred to Petri dishes with pest-free feed obtained from previous research. Larvae obtained from stomach content were examined with the same method. The developmental forms of the insects were identified under the Leica M165C stereoscopic microscope (7.2x–120x magnification). Image acquisition of the discovered larvae were performed in the Leica Application Suite program. The description published by Bakuła [3] was used to identify the insect species.

## Data Availability

The datasets used during the current study are available from the corresponding author on reasonable request.

## Abbreviations

*T. molitor*: *Tenebrio molitor*
IgE: Immunoglobulin E
PRRS: Porcine Reproductive and Respiratory Syndrome
b.w.: body weight
PCV2: porcine circovirus 2
